# Vimentin gets a new glow from zinc

**DOI:** 10.18632/oncotarget.4649

**Published:** 2015-06-25

**Authors:** Dolores Pérez-Sala, Clara L. Oeste, Francisco J. Sánchez-Gómez

**Affiliations:** Dept. of Chemical and Physical Biology, Centro de Investigaciones BIológicas, C.S.I.C., Madrid, Spain

**Keywords:** Chromosome Section, vimentin dynamics, cysteine oxidation, zinc binding, zinc deficiency

Intermediate filaments are important beams in cell architecture. Keratin filaments, neurofilaments, the constituents of the nuclear lamina and the vimentin network are examples of these structures, which confer mechanical resistance to cells and at the same time are flexible and dynamic. Vimentin is an intermediate filament protein expressed in mesenchymal cells. Leading-edge research in the last decade has shown that besides its structural purpose, vimentin plays other important roles in cell homeostasis, including promoting the correct position and function of organelles, the interaction with and regulation of signaling proteins, and a bidirectional cross-talk with other cytoskeletal structures [1, 2]. In addition, vimentin plays a leading role in numerous pathophysiological processes including epithelial mesenchymal transition, infection, autoimmune disease, and wound healing.

In spite of many years of excellent research in this field [1, 3], vimentin still conceals some secrets. The structure of this coiled-coil protein has not been fully characterized and its mode of assembly in cells is incompletely understood. Although many stimuli have been identified that promote vimentin disassembly, less is known about the induction of vimentin filaments polymerization or elongation. Thus, in some aspects, vimentin is still a mysterious protein, compared to the building blocks of other cytoskeletal structures such as tubulin in microtubules or actin in microfilaments.

This 55 kDa protein possesses a single cysteine residue, cys328. In recent work we have shown that this residue is a remarkable sensor for oxidative stress [4]. Mutating this residue numbs the responsiveness of the vimentin network to oxidants and electrophilic compounds, attenuating its remodeling and making it somehow “resistant” to these aggressions [4, 5]. However, this mutant does not keep up with vimentin's demanding tasks in various cellular processes and shows limited performance in the expansion of the vimentin network after cell plating, in achieving the appropriate distribution of cytoplasmic organelles such as lysosomes, and in forming aggresomes upon inhibition of proteasomal degradation or extensive oxidative damage.

While studying the mechanisms underlying the requirement for cys328 for optimal vimentin function, we found that vimentin binds zinc [4]. We think that this is an unprecedented, exciting finding for this protein, which may turn the spot-light on some previously dim features in this field.

Zinc is the transition metal most frequently found as a cofactor in proteins [6]. Zinc binding to proteins may serve regulatory or structural roles, either by promoting specific conformations, sustaining catalytic activity or stabilizing oligomeric associations.

In vitro, vimentin avidly binds zinc with high affinity, and submillimolar zinc reversibly promotes vimentin polymerization inducing the formation of vimentin bundles. Zinc binding to vimentin is suprastoichiometric, which suggests that it involves multiple electrostatic interactions likely due to the polyelectrolyte nature of the protein [3].

In cells, zinc availability has a strong impact on vimentin function. Zinc reversibly regulates the assembly of fluorescent GFP-vimentin constructs in vimentin-deficient cells. Moreover, whereas pharmacological zinc chelation alters the vimentin network, genetic zinc deficiency is associated with increased vimentin susceptibility to further zinc depletion or oxidative stress, together with a lower proportion of polymerized vimentin and a thinner appearance of filaments, and these alterations are alleviated by zinc supplementation. Interestingly, cellular imaging with the fluorescent zinc probe, zinquin, lights-up portions of vimentin bundles, indicating that zinc interacts with vimentin in cells. Although the interaction of zinc with other cytoskeletal proteins has been reported, the affinity of vimentin appears to be much higher based on the behavior of the purified proteins. If such an efficient binding takes place in cells, vimentin could behave as a zinc reservoir, or as a zinc-sequestering agent. Moreover, the stimulating possibility exists that the interaction between zinc and vimentin has reciprocal outcomes, that is, zinc may regulate vimentin, but vimentin could also play an important role in cellular zinc homeostasis. In light of our findings it would be tempting to speculate that vimentin can fulfil this role through a direct interaction with zinc.

**Figure 1:**
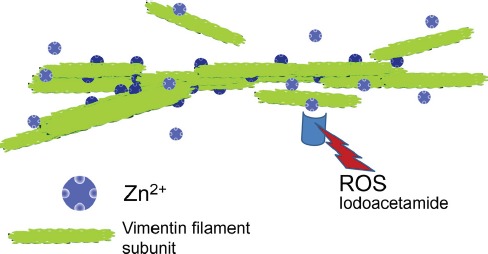
Vimentin subunits associate to form filaments Zinc may stabilize this association and shield the protein from oxidative stress.

Although the precise structural features of the vimentin-zinc interaction have not been elucidated, we may picture zinc atoms as staples that reversibly stabilize filaments at specific places. Zinc can be incorporated into proteins in Zn-clusters, often of tetrahedral geometry, in which a zinc atom is coordinated by four ligands, most frequently involving sulfur from cysteine, nitrogen from histidine and/or oxygen from carboxylic residues [7]. In addition, zinc could interact with vimentin through electrostatic interactions, as it has been described for other divalent cations such as calcium or magnesium [3]. These possibilities are not mutually exclusive. The fact that the cys328 vimentin mutant appears to be resistant to zinc-deficiency indicates that this residue plays a role in the effects of zinc through direct or indirect mechanisms. Besides the complex interactions between zinc binding and redox regulation of cysteine residues, zinc may affect overall cellular status by interacting with transcription factors or other zinc binding proteins.

The pathophysiological implications of these findings are far-reaching. Besides the broad implications of vimentin in disease, zinc deficiency is extremely frequent, due to various pathologies but, above all, to insufficient dietary intake. Symptoms of this condition may include dermatitis, impaired wound healing, diarrhea, and cognitive alterations. Our findings raise the possibility that defective vimentin, or hypothetically, intermediate filament function, may underlie some of these alterations [4]. Therefore, we believe that they introduce a new perspective from which to revisit some concepts in this field.
